# Internalization of miPEP165a into *Arabidopsis* Roots Depends on both Passive Diffusion and Endocytosis-Associated Processes

**DOI:** 10.3390/ijms21072266

**Published:** 2020-03-25

**Authors:** Mélanie Ormancey, Aurélie Le Ru, Carine Duboé, Hailing Jin, Patrice Thuleau, Serge Plaza, Jean-Philippe Combier

**Affiliations:** 1 Laboratoire de Recherche en Sciences Végétales, UMR5546, Université de Toulouse, UPS, CNRS, 31320 Auzeville-Tolosan, France; carine.duboe@lrsv.ups-tlse.fr (C.D.); thuleau@lrsv.ups-tlse.fr (P.T.); serge.plaza@lrsv.ups-tlse.fr (S.P.); 2 Fédération de Recherche FR3450, CNRS, Université de Toulouse, 31326 Castanet-Tolosan, France; leru@lrsv.ups-tlse.fr; 3 Department of Microbiology & Plant Pathology, Center for Plant Cell Biology, Institute for Integrative Genome Biology, University of California, Riverside, CA 92521, USA; hailing.jin@ucr.edu

**Keywords:** *Arabidopsis*, endocytosis, microRNAs, miPEPs, peptides

## Abstract

MiPEPs are short natural peptides encoded by microRNAs in plants. Exogenous application of miPEPs increases the expression of their corresponding miRNA and, consequently, induces consistent phenotypical changes. Therefore, miPEPs carry huge potential in agronomy as gene regulators that do not require genome manipulation. However, to this end, it is necessary to know their mode of action, including where they act and how they enter the plants. Here, after analyzing the effect of *Arabidopsis thaliana* miPEP165a on root and aerial part development, we followed the internalization of fluorescent-labelled miPEP165a into roots and compared its uptake into endocytosis-altered mutants to that observed in wild-type plants treated or not with endocytosis inhibitors. The results show that entry of miPEP165a involves both a passive diffusion at the root apex and endocytosis-associated internalization in the differentiation and mature zones. Moreover, miPEP165a is unable to enter the central cylinder and does not migrate from the roots to the aerial part of the plant, suggesting that miPEPs have no systemic effect.

## 1. Introduction

Gene expression is the consequence of the transcription of an RNA molecule from a gene—modulated by transcription factors and modifications of the chromatin structure—and post-transcriptional mechanisms acting on the RNA stability of translation or on the protein it encodes. One of the best-known mechanisms of post-transcriptional regulation of gene expression is gene silencing induced by microRNAs (miRNAs). MiRNAs are small, regulatory RNA molecules (21–24 nucleotides) first discovered in the worm *Caenorhabditis elegans* and later in plants and humans [1,2,3,4]. Each miRNA regulates the expression of specific target gene(s) either by cleaving the mRNA transcribed from it or by inhibiting its translation. Target genes of miRNAs are often key regulatory genes encoding, for example, transcription factors or hormone receptors. MiRNAs are therefore required for the correct regulation of most developmental processes in plants and animals, and dysregulation of miRNA expression is a feature of many human pathologies.

MiRNAs are themselves encoded by genes and are transcribed in the form of long primary transcripts (pri-miRNAs). One of the first steps in the maturation of pri-miRNAs involves a nuclear protein complex containing an enzyme called dicer-like 1 (DCL1), which cleaves pri-miRNAs to form precursor miRNAs (pre-miRNAs). A second cleavage step then forms mature miRNAs. In the cytoplasm, the mature miRNA anneals by homology with the mRNA of its target gene(s). This heteroduplex molecule is recognized by a protein complex called RISC, containing the enzyme Argonaute (AGO1), which either cleaves the targeted mRNA or inhibits its translation. Because the main role of miRNAs is to act as regulatory small RNAs and not in the direct translation of proteins, miRNAs have always been thought to be non-coding RNAs.

Surprisingly, the characterization of plant pri-miRNAs revealed that they encode small regulatory peptides, which were called miPEPs for miRNA-encoded peptides [5]. MiPEPs are involved in a positive autoregulatory feedback loop. They specifically activate transcription of their primary transcript and consequently enhance the synthesis of the mature miRNA, thus turning down the expression of specific genes. Interestingly, the application of exogenous synthetic miPEPs to plants is sufficient to stimulate the synthesis of their corresponding miRNAs and to modify plant development accordingly [6,7]. Given their efficiency simply by an external application on plants, miPEPs are promising molecules for many agronomic applications. In particular, they offer a new way of modulating plant development, stimulating plant symbioses, or increasing plant fitness, to name a few potential uses. Moreover, as natural and endogenous peptides, they are likely to be much less harmful to the environment than chemical treatments and more acceptable to the general public than genetically modified organisms. 

Endocytosis plays a crucial role in the internalization of extracellular molecules and plasma membrane proteins into eukaryotic cells [8]. Clathrin-mediated endocytosis (CME) remains the most extensively studied and characterized endocytosis and constitutes the major route of entry and pathway in eukaryotes [8,9]. Clathrin is a triskelion-shaped scaffold protein composed of three clathrin light chains (CLCs) and three clathrin heavy chains (CHCs). The formation of clathrin-coated vesicle at the plasma membrane requires adaptor proteins, including AP2 complex [10]. In plants, CME is involved in multiple important biological processes, including growth, development, nutrient uptake, and biotic and abiotic stress responses [8,10,11,12,13,14,15]. For instance, clathrin is required for plasma membrane-located receptor endocytosis upon peptide perception, leading to peptide-mediated responses and thus to plant immunity [15,16]. Moreover, recent studies have also reported the existence of sterol-sensitive clathrin-independent pathways in plants, although this alternative endocytosis pathway is far less understood [17,18]. The best-studied clathrin-independent pathway in plants corresponds to flotillin-1-mediated endocytosis, a membrane microdomain-associated protein involved in plant development and promoted by flg22, a flagellin-derived 22-amino acid peptide [19,20]. Alternatively, proteins can assemble into clusters in membrane microdomains [8]. For instance, remorins form clusters at the plasma membrane and interact with a symbiotic receptor that allows bacterial infection in *Medicago truncatula* [21]. Finally, both clathrin-dependent and -independent pathways can be constitutive or differentially regulated in response to stimuli [17,18,22]. In summary, different endocytosis pathways have been reported to be involved in many biological outcomes. 

Due to their capacity to modulate plant development, miPEPs are of interest in agronomy as an alternative to chemicals to stimulate plant development. Nevertheless, to achieve this goal, a better understanding of their mode of action at the molecular level, including the mechanisms of their entry into plants, is required. In this study, we investigated how miPEPs enter into plants. We first reported in detail the phenotypes observed after treatment of *Arabidopsis thaliana* with miPEP165a, previously used to decipher the mode of action of miPEPs [5]. By using this miPEP labelled with a fluorescent dye, we followed the internalization of the peptide into plants. The peptide entered rapidly into the root cap and the meristematic zone and it took longer to penetrate the other parts of the root. Using mutants potentially altered in endocytic pathways or chemical inhibitors affecting endocytosis, we identified two mechanisms of miPEP165a entry into roots, passive diffusion followed by an endocytosis process.

## 2. Results

### 2.1. MiPEP165a Promotes Cell Division in the Meristematic Zone to Increase Primary Root Length and Acts on Flowering Time in Arabidopsis

**Figure 1:**
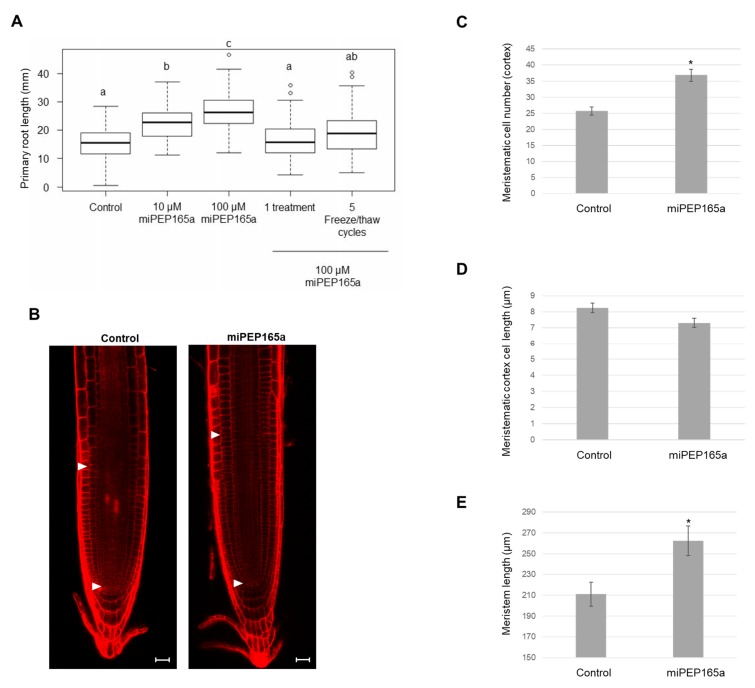
MiPEP165a promotes root growth by enhancing cell division. (**A**) Effect of miPEP165a on primary root length. *Arabidopsis thaliana* seedlings were treated with various concentrations of miPEP165a. Seedlings were treated daily with the peptide for 4 days, with the exception of those that received only one treatment. Peptides were thawed once, except those that underwent five freeze/thaw cycles. Two-way analysis of variance (ANOVA) significance levels were based on Tukey’s post-test (1-way ANOVA), (a–c, *p* < 0.05, *n* = 70). At least three biological replicates were performed (**B**–**E**). Three-day-old seedlings were treated daily with water or 100 µM miPEP165a for a further 3 days and stained with 10 µg/mL propidium iodide for 20 min. (**B**) Confocal images showing the meristematic zone for the cortex cells, defined as the region between quiescent center cells and the first elongating cell that was twice the length compared to its distal neighbor (distance between white arrows) [25,26]. Meristematic cell number (**C**) and cell length (**D**) were determined with the software tool Cell-o-Tape, an open source ImageJ/Fiji macro [27,28,29]. (**E**) Quantification of root apical meristem length. (**B**–**E**) Four biological replicates were performed with at least 20 seedlings. Errors bars represent SEM. Asterisks indicate a significant difference at *p* < 0.01 (*) according to the t-test. Scale bar = 25 µm. Water was used as a control.

It has been previously shown that *A. thaliana* miPEP165a, as well as miR165a, is expressed in endodermis cells [5,23]. Exogenous treatment of *A. thaliana* seedlings with synthetic miPEP165a is sufficient to increase the primary root length [5]. However, the precise mechanisms (spatial and temporal) involved in the peptide uptake remained unknown. To study the entry of miPEPs, especially miPEP165a, we first defined the best experimental conditions to obtain a significant effect of miPEP165a on plant development. We first observed that watering plants with 100 µM of peptide was much more efficient at increasing the primary root length than treatments performed with only 10 µM of peptide, the concentration used in the previous study [5] (Figure 1A). In addition, similar to the concentration of 10 µM previously used [5], applying miPEP165a at 100 µM also induced the activation of the pri-miRNA from which it originates (Figure S1A). In addition, during the initial stages of the study, when the effect of miPEP165a on primary root length was studied, whatever the control used, i.e., scrambled miPEP165a, irrelevant peptides, or their corresponding solvents (acetonitrile or water), no response was observed compared to miPEP165a treatments (Figure S1B). Similarly, water and scrambled miPEP165a had no effect on the expression of pri-miR165a compared to miPEP165a (Figure S1A). Finally, we observed that several freeze/thaw cycles of the peptide were detrimental to its activity on the length of primary roots (Figure S1C). For these reasons, we used aliquots of unfrozen peptides only once and kept water as a reference in all the following experiments. 

The increase in root length upon treatment may be a consequence of higher cell elongation or increased cell proliferation. To address this point, we analyzed the effect of miPEP165a at the cellular level on the meristematic zone since root growth was often determined by meristematic activity [24]. We revealed that more cells were present in the meristematic zone when roots were treated with miPEP165a (Figure 1B–E). Therefore, these experiments suggest that the increase in root length induced by the miPEP165a treatment is likely due to the stimulation of cellular proliferation rather than an increase in cell length.

**Figure 2:**
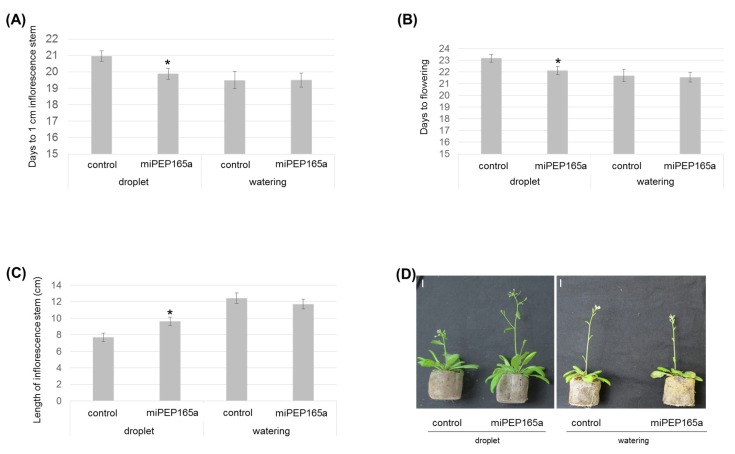
Flowering phenotypes of *Arabidopsis* plants in response to miPEP165a treatment. *Arabidopsis* plants were treated with either water (control) or a droplet of 100 µM miPEP165a placed on the shoot apical meristem or by watering with 10 µM miPEP165a three times a week until analyses. Flowering time measurements were determined using the number of days to obtain an inflorescence stem of 1 cm (**A**) and the number of days to obtain the first flowers (**B**). (**C**) The length of the *Arabidopsis* inflorescence stem was determined 24 days after sowing. Error bars indicate SEM. Statistical analysis was performed using a *t*-test (*p* < 0.01). (**D**) Representative pictures showing the flowering phenotype according to the miPEP165a treatment. Experiments were performed at least 4 independent times (*n* > 78 plants). Bar = 1 cm.

MiR165a and its target genes, *REVOLUTA* (*REV*), *PHABULOSA* (*PHB*), and *PHAVOLUTA* (*PHV*), are also known to be involved in flowering [30]. To investigate whether miPEP165a could have an effect on flowering, we treated the shoot apical meristem with a droplet of 100 µM miPEP165a three times a week during plant development. Treatments with miPEP165a accelerated plant development as illustrated by the decrease of the flowering day (Figure 2A,B) and the increase of the length of the inflorescence stem (Figure 2C,D). Interestingly, watering the roots with 10 µM peptide had no effect on the flowering, suggesting that peptides cannot migrate throughout the plant (Figure 2). 

### 2.2. MiPEP165a Entry Involves both Passive Diffusion at the Root Apex and Endocytic Pathways in the Differentiation and Mature Zones 

**Figure 3:**
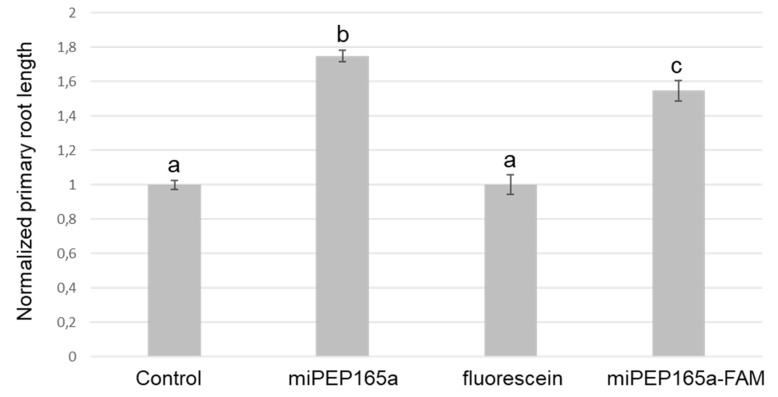
MiPEP165a-FAM is biologically active. Seedlings were treated with water (control), 100 µM miPEP165a, miPEP165a-FAM, or fluorescein. At least 70 seedlings were used to determine the normalized *Arabidopsis* root length. Data are given as ± SEM and statistical analysis was performed using a t-test (a–c, *p* < 0.01).

To document this observation, we used the miPEP165a labelled with FAM, a fluorescent dye derived from fluorescein. As illustrated in Figure S2, the physiochemical properties of the miPEP165a-FAM are similar to those of the non-modified peptide. Although slightly less active, the labelled peptide was still able to increase the primary root length (Figure 3).

**Figure 4:**
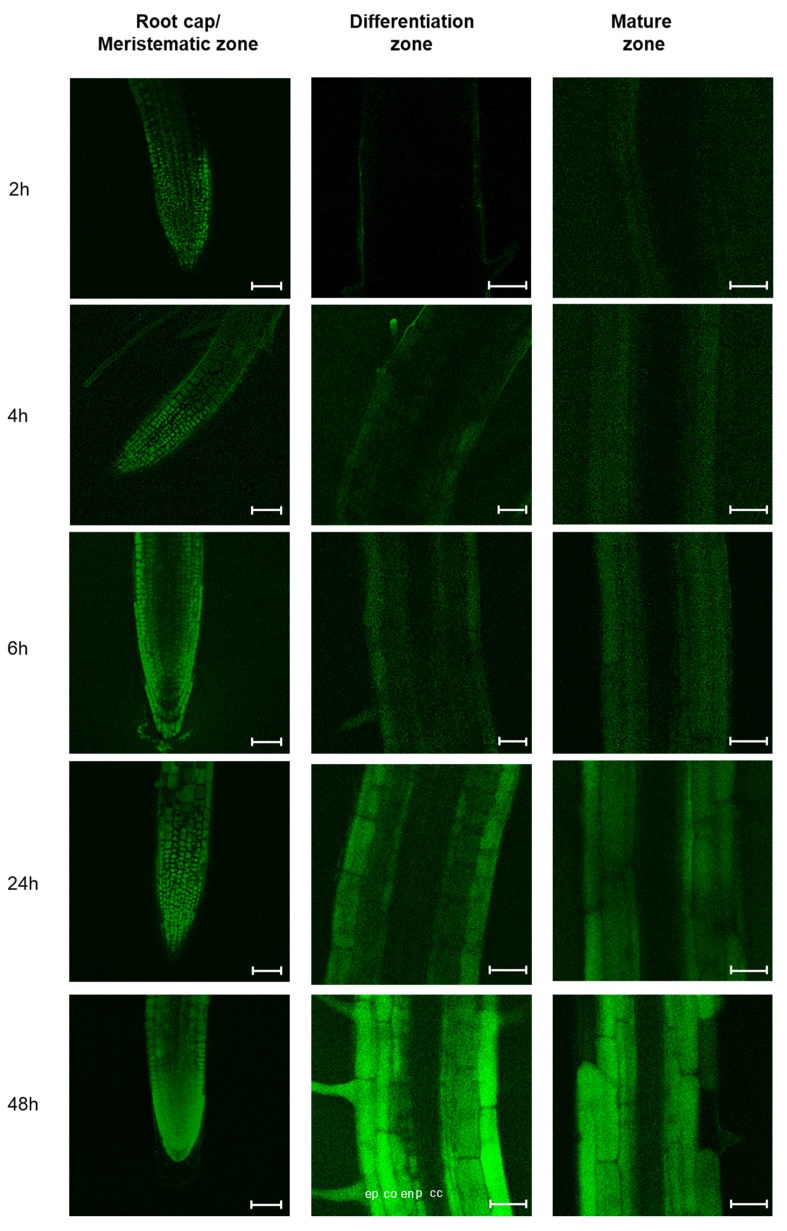
Kinetics of miPEP165a uptake into *Arabidopsis* roots. The mobility of miPEP165a-FAM was followed at the indicated time in different zones of *Arabidopsis* roots, as defined by [31]. Confocal images are representative of four independent experiments, with at least 6 seedlings for each condition. Bar = 50 µm (root cap/meristematic zone) or 25 µm (differentiation and mature zones). The different cell layers are indicated in the differentiation zone image at 48 h as follows: cc, central cylinder; p, pericycle; en, endodermis; co, cortex; ep, epidermis.

Interestingly, while the labelled peptide penetrated rapidly (~2 h) into the root cap and the meristematic zone, it took longer to penetrate the other parts of the root (Figure 4). Twenty-four hours after the application of the labelled peptide, the latter was present in most external parts of the roots. The central cylinder was never labelled by the peptide, which seemed to be blocked by the pericycle (Figure 4). 

**Figure 5:**
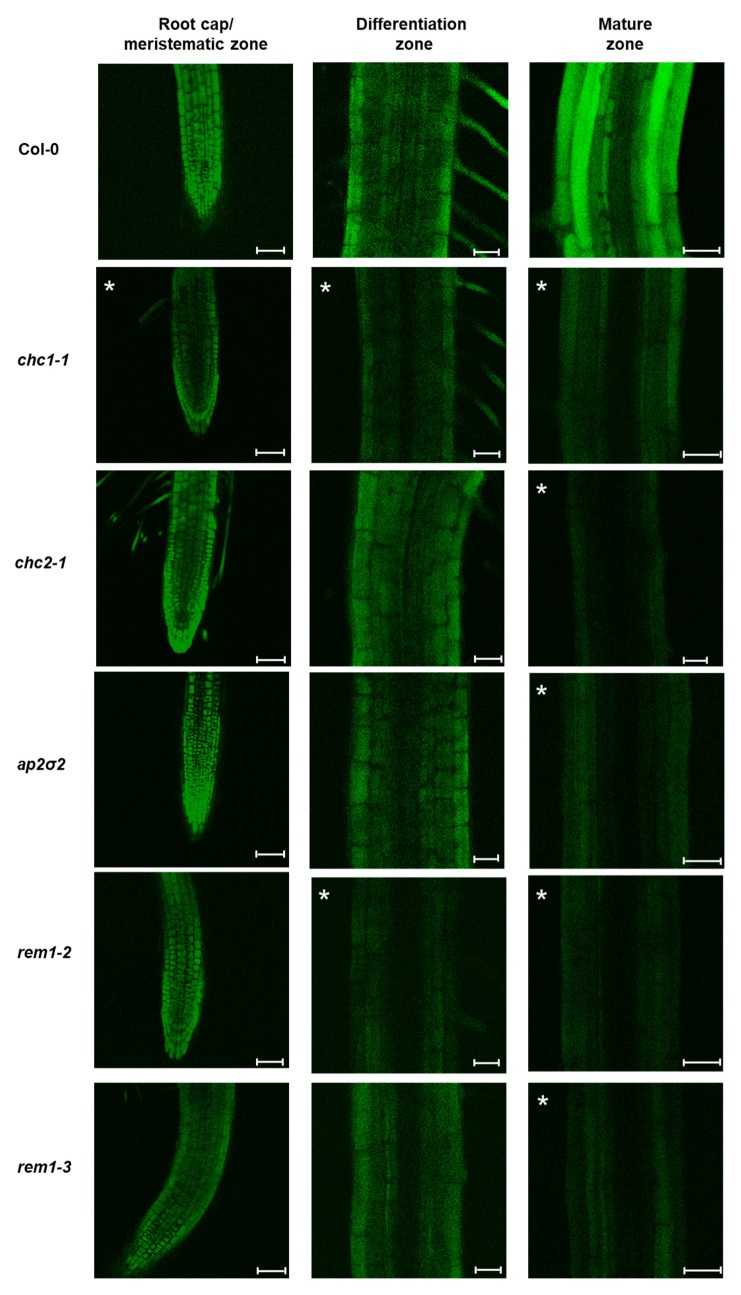
Internalization of miPEP165a is clathrin and remorin dependent. Representative confocal images showing the uptake of miPEP165-FAM 48 h after treatment in wild-type seedlings and *chc1-1*, *chc2-1*, *ap2σ2*, *rem1-2*, and *rem1-3* mutants. A significant fluorescence decrease for each condition is indicated in each panel by asterisks. Quantifications of the fluorescence intensity from more than 15 seedlings are shown in Figure S3. Bar = 50 µm (root cap/meristematic zone) or 25 µm (differentiation and mature zones).

The entry of peptides into plants might occur passively, by diffusion, or actively, via specific transporters or by endocytosis. Because of the huge diversity of miPEPs in a plant and the lack of conservation between species [5], we hypothesized that specific transporters for each peptide are unlikely to exist and, more likely, the miPEPs might be internalized by generic internalization machinery or, more simply, by passive diffusion. To decipher the mechanisms involved in the entry of peptides into cells, we used *A. thaliana* mutants impaired on genes encoding proteins associated to the clathrin pathway (*chc1-1*, *chc2-1*, *ap2σ2*) [12,16,32] or to the membrane microdomain (*rem1-2*, *rem1-3*) [33,34,35]. Internalization of miPEP165a was not affected in most of the mutants tested, except in the root cap/meristematic zone of the *chc1-1* mutant and in the differentiation zone of the *chc1-1* and *rem1-2* mutants, suggesting that uptake in these parts was mainly passive (Figure 5, Figure S3). Conversely, the entry of the peptide into the mature zone of all mutants was strongly impaired (Figure 5, Figure S3). These data suggest that peptide entry in plants involves, in addition to passive diffusion, both clathrin and membrane microdomain-mediated pathways.

**Figure 6:**
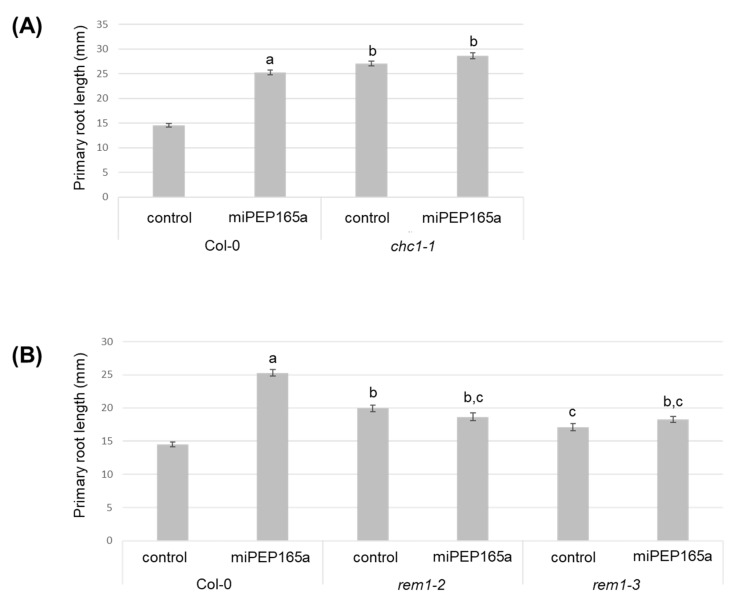
MiPEP165a-mediated root growth induction involves clathrin and remorin proteins. Measurement of the primary root length in *chc1-1* (**A**) and remorin (*rem1-2* and *rem1-3*) mutants (**B**) after water (control) or miPEP165a (100 µM) treatment. The error bars indicate SEM of at least three biological replicates (n > 110 seedlings) and statistical analyses were performed using a t-test (a–c, *p* < 0.01).

In order to determine how and to what extent a defect in the peptide entry affects its biological effect on plant development, we treated the roots of *chc1-1*, *rem1-2*, and *rem1-3* mutants with the peptide in parallel with the wild-type roots. While the mutants showed a longer primary root in the control conditions compared to the wild type plants, they were unable to respond to the peptide by increasing their primary root length (Figure 6). Indeed, the *rem1-2* mutant, which was strongly affected in the peptide uptake, was unable to respond to miPEP165a. 

**Figure 7:**
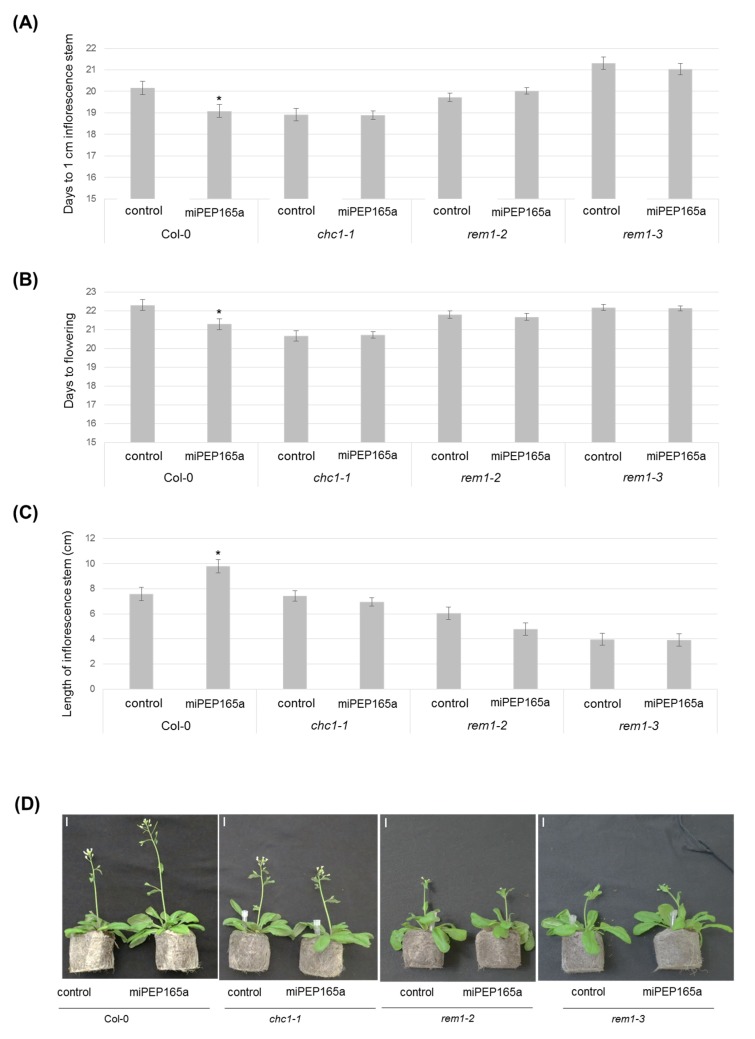
Flowering time depends on clathrin- and membrane microdomain-associated pathways. The number of days to obtain a 1-cm inflorescence stem (**A**) and the number of days to observe the first flowers (**B**) were determined for wild-type plants as well as for *chc1-1*, *rem1-2*, and *rem1-3* mutant plants. (**C**) Measurement of the inflorescence stem length was determined 24 days after sowing for wild-type and mutant plants. Data are representative of the average of at least four independent experiments with at least 10 plants per condition, for each experiment. Error bars represent SEM and statistical analyses were performed using a t-test (*, *p* < 0.01). (**D**) Representative images comparing wild-type and mutant plants treated with water (control) or miPEP165a. Bar = 1 cm.

We next treated the aerial parts of the mutants with miPEP165a, and we observed similar results on the flowering time (Figure 7). These results suggest that the mechanisms of miPEP165a uptake into roots and aerial parts could be similar. 

**Figure 8:**
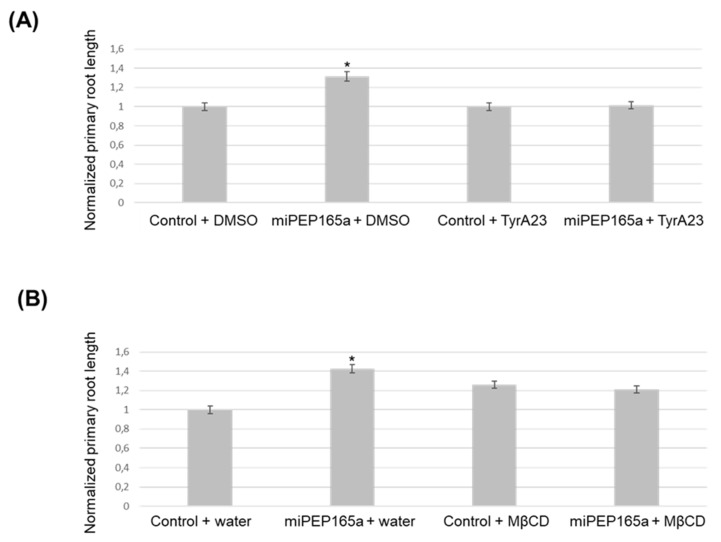
Disruption of endocytic pathways prevents miPEP165a-induced root growth. Normalized primary root growth analysis after treatment with miPEP165a and TyrA23 (**A**) or miPEP165a and MβCD (**B**). Three biological replicates were performed by using at least 100 seedlings for each condition and root lengths were statistically analyzed using a *t*-test (*p* < 0.01, *). The data represent the mean value ± SEM. Water was used as a control for the miPEP165a treatment.

Finally, we used TyrA23, a chemical inhibitor known to affect clathrin-mediated endocytosis [22,32,36], and MβCD, a cholesterol-depleting agent, which have been suggested to block microdomain-dependent endocytosis [17,18,22]. Interestingly, both molecules were able to inhibit the miPEP165a-activated root length phenotype, suggesting that peptide entry in plant involves clathrin-mediated endocytosis and membrane microdomain-dependent pathways (Figure 8).

Altogether, our results showed that miPEP165a entry used passive diffusion at the root apex followed by endocytosis in the differentiation or mature zone of plant roots. All pathways are required to mediate full peptide uptake (and activity).

## 3. Discussion

MiRNAs have been considered for a long time as non-coding RNAs. However, a few years ago, it was shown that pri-miRNAs can encode regulatory peptides, which were named miPEPs. These miPEPs activate the transcription of their associated miRNA and thus downregulate the expression of their target genes [5]. Among miPEPs, miPEP165a induces the accumulation of mature miR165a, known to repress the expression of all five class III homeodomain-leucine zipper (HD-ZIP III) transcription factors, i.e., *REV*, *PHB*, *PHV*, *CORONA* (*CAN/AtHB15*), and *AtHB8* [5,37]. In *Arabidopsis*, the overexpression of all HD-ZIP III results in plants with shorter roots whereas *phb, phv* double mutants and *phv-11* mutants display longer roots as well as an increase in the number of meristem cells compared to wild-type plants [38,39]. Moreover, the overexpression of miR166, differing by only one nucleotide from miR165 and targeting the expression of three HD-ZIP III genes, also promotes primary root growth in *Arabidopsis* [39]. These results can be correlated with those of the present study, since we showed that miPEP165a promotes primary root growth by increasing cell division in the root apical meristem (Figure 1). Moreover, misexpression of the HD-ZIP III genes by making them resistant to miR165/166 and a reduction in the expression of HD-ZIP IIIs by overexpression of miR165/166 induces prolonged activity of floral stem cells [30]. Here, we observed that miPEP165a accelerates the appearance of the inflorescence stem and the flowering time of *Arabidopsis* wild-type plants (Figure 2).

Since some small peptides were considered as long-distance signaling molecules, we wondered whether miPEP165a was involved in root/shoot communication [40,41,42]. By tracking the FAM-labelled miPEP165a across all layers of *Arabidopsis* roots, we showed that the labelled peptide entered into the epidermis and migrated up to the pericycle but did not reach the root vessels (Figure 4). Moreover, the acceleration of flowering observed in response to the miPEP165a treatment of the shoot apical meristem was not observed after watering *Arabidopsis* roots with miPEP165a (Figure 2). Taken together, these results indicate that miPEP165a is not a root-to-shoot mobile signal molecule. 

Consequently, in order to have a better understanding of miPEP uptake into plants, we investigated the mobility of FAM-labelled miPEP165a in *Arabidopsis* roots. Clathrin-mediated endocytosis is the major and the most studied route of entry in plants [8]. A recent study showed that this endocytic pathway is necessary for the internalization of the elicitor peptide *At*pep1 and its receptor, leading to *At*pep1-induced responses [16]. Here, we showed that the entry of miPEP165a could also be dependent on clathrin since miPEP165a uptake was significantly decreased in the primary roots of *chc1-1*and strongly reduced in the mature zone in the three mutants *chc1-1*, *chc2-1*, and *ap2σ2* (Figure 5). These results were confirmed by the fact that the increase of the root length by miPEP165a was not observed in the *chc1-1* mutant or after treatment with TyrA23 (Figure 6A, Figure 8A), the most commonly used CME inhibitor [8,32,36]. Similarly, the acceleration of the flowering time induced by miPEP165a in wild-type plants was not observed in the *chc1-1* mutant (Figure 7).

Besides clathrin-mediated endocytosis, membrane microdomain-associated endocytosis has been described in plants as an alternative route of entry pathway [8]. This endocytosis pathway is sensitive to sterol depletion and consequently to the sterol-depleting agent MβCD [8,17,18]. In the present study, we showed that MβCD prevented miPEP165a-FAM entry and correlatively the increase of root length induced by miPEP165a (Figure 8B, Figure S4). Collectively, our results indicate that both clathrin-dependent pathways and microdomain-associated events may cooperate in peptide entry into *Arabidopsis* roots. Previous results have demonstrated that internalization of the aquaporin PIP2;1 and RbohD involved both dependent and independent clathrin-mediated endocytosis, the latter being stimulated in saline stress conditions [17,22]. Stimulation of the endocytic pathway under salt stress requires the simultaneous action of both clathrin-dependent and membrane microdomain-associated endocytosis [17,22]. In addition, Baral and his colleagues have shown that clathrin-mediated endocytosis allows the internalization of transmembrane proteins in all cell root layers whereas a sterol-sensitive clathrin-independent pathway internalizes lipid-anchored cargoes only in the epidermal cell layer [18]. Moreover, these authors showed that salt stress activates an additional clathrin-independent endocytosis pathway across all cell root layers that takes up both molecule types [18]. Considering membrane microdomain-associated endocytosis, it is known that proteins assemble into clusters in lipid rafts [8]. Among these proteins, remorins are considered as markers of membrane microdomains [35]. In *Medicago truncatula*, the symbiotic remorin 1 forms clusters and interacts with symbiotic receptors at the plasma membrane, playing a key role in bacterial signal perception [21]. Here, we showed that remorins 1-2 and 1-3, which are among the 10% of the most highly expressed genes in *Arabidopsis* [43], were also involved in miPEP165a entry into *Arabidopsis* roots (Figure 6B, Figure 7). Indeed, miPEP165a-FAM failed to enter the differentiation zone of *Arabidopsis* roots in *rem1-2* and *rem1-3* mutants. Moreover, root length and flowering acceleration induced by miPEP165a were perturbed in both remorin mutants (Figure 6B, Figure 7). 

To conclude, we showed that endocytic pathways participate in miPEP uptake in plants. Thus, clathrin-mediated endocytosis as well as membrane microdomain-associated pathways seem to cooperate, allowing miPEPs to regulate their corresponding miRNAs and consequently modulate the plant phenotype, such as flowering and root development. Due to the simplicity of the mode of administration of miPEPs, a better understanding of miPEP uptake into plants is a first step towards the possible agronomic application of peptides. 

## 4. Materials and methods

### 4.1. Peptide Synthesis

miPEP165a (MRVKLFQLRGMLSGSRIL), miPEP165a fused to fluorescein (miPEP165a-FAM), scrambled miPEP165a (LMGRQGLKISSLVFRMLR), PEP1 (KSNKTRVNFPS), PEP2 (MCFSFPDL), and PEP3 (MASAAKVYMA) were synthetized by Smart Bioscience (https://www.smart-bioscience.com/). They were dissolved in water (control) as a 10 mM stock solution (except for PEP2, which was dissolved in 50% acetonitrile as a 2 mM stock solution), aliquoted, and conserved at −80 °C until use. 

### 4.2. Plant Materials 

Different *Arabidopsis thaliana* plant lines (Columbia Col-0 ecotype) were used: the *chc1-1* (At3g11130), *chc2-1* (At3g08530), *ap2σ2* (At1g47830), *rem1-2* (At2g45820), and *rem1-3* (At3g61260) *Arabidopsis* mutants.

### 4.3. Peptide Treatment of Arabidopsis Roots

Surface-sterilized *Arabidopsis* seeds were sown on the surface of cellophane membrane placed on ½ MS solid medium and stratified for one day at 4 °C in the dark. Seeds were vertically grown in controlled environmental chambers at 22/20 °C, with a photoperiod of 16h light/8h dark, an irradiance of ~ 97.5 µmol photons.m^−2^.s^−1^, and a relative humidity of 40%. Three days after sowing, seedlings were treated daily for 4 days either with water, 2.5% acetonitrile, 100 µM scrambled miPEP165a, 100 µM irrelevant peptides (PEP1, PEP2, PEP3), or fluorescein (control conditions) or with 100 µM miPEP165a or miPEP165a-FAM (treated conditions). Twenty-four hours after the last treatment, seedlings were scanned in order to measure primary root lengths using NeuronJ plugin of ImageJ. 

### 4.4. Peptide Uptake in Arabidopsis Roots

Surface-sterilized wild-type and mutant *Arabidopsis* seeds were grown onto ½ MS solid medium in the same conditions as those described in the previous section. Three days after germination, three seedlings were transferred to each well of a 48-well plate containing 200 µL of ½ MS liquid medium. One day later, medium was replaced by 10 µM miPEP165a-FAM diluted in ½ MS liquid medium until confocal microscopy observations. FAM fluorescence was analyzed with a confocal laser scanning microscope (Leica TCS SP2-AOBS using a 40 X water immersion objective lens (numerical aperture 0.80; HCX APO). FAM fluorescence was excited with the 488-nm ray line of the argon laser and recorded in the 511–551-nm emission range. 

For quantification of miPEP165a-FAM entry into wild-type and mutant *Arabidopsis* roots, the fluorescence intensity was determined per surface unit in the different root zones using ImageJ software.

### 4.5. Inhibitor Treatment 

TyrA23 was dissolved in dimethyl sulfoxide to yield a 50 mM stock solution and MβCD was prepared in deionized water at a final concentration of 38 mM. For each experiment, 3-day-old seedlings germinated on ½ MS solid medium + 1% sucrose (wt/vol) were pre-treated with 50 µM TyrA23 or 10 mM MβCD for 30 min [17]. Seedlings were then treated with the inhibitors supplemented with 100 µM miPEP165a. Treatments were performed daily for an additional 3 days and plates were scanned for analysis of the primary root length with NeuronJ, an Image J plugin [29,44].

### 4.6. Flowering Phenotype 

*Arabidopsis* seeds were grown on Jiffy^®^ under a 16 h light/8 h dark cycle (22/20 °C), with a relative humidity of 80%. Fifteen days after seed sowing, either a 2-µL droplet of 100 µM miPEP165a was put on the shoot apical meristem or seedlings were watered with 500 µL of 10 µM miPEP165a three times a week. Analyses of the aerial parts were performed 24 days after sowing. 

### 4.7. Propidium Iodide Staining

Wild-type seeds were grown for 3 days on ½ MS solid medium + 1% sucrose (wt/vol) in the same growth conditions as described above. Seedlings were then treated with water or 100 µM miPEP165a daily for 3 additional days and placed in the growth chamber at the same settings. Seedlings were then stained with 10 µg/mL propidium iodide for 20 min and *Arabidopsis* cell roots were analyzed with a laser scanning confocal microscope (Leica TCS SP8-AOBS) with a ×25 water immersion objective lens (numeral aperture 0.95; Fluotar Visir). The excitation and emission wavelengths of propidium iodide were 561 and 570–640 nm, respectively.

The meristematic zone for the cortex cells was defined as the region between quiescent center cells and the first elongating cell that was twice the length compared to its distal neighbor [20,26]. The meristematic cell length and cell number were determined with the software tool Cell-o-Tape, an open source ImageJ/Fiji macro [27,28,29]. At least 20 roots were analyzed for each treatment.

### 4.8. Immunoblots and RT-qPCR

Seven-week-old *Arabidopsis* seedlings were treated with 100 µM miPEP165a or its corresponding control for 24 h, and then the expression of pri-miR165a was evaluated by RT-qPCR according to Lauressergues et al. [5]. 

To evaluate miPEP165a stability, 5 nanomoles of miPEP165a were subjected to several freeze/thaw cycles and its degradation was detected by immunoblotting with an anti-miPEP165a antibody as previously described [5].

## Supplementary Materials

The following are available online at https://www.mdpi.com/1422-0067/21/7/2266/s1, Figure S1. Effect of miPEP165a and importance of its stability. Figure S2. Physiochemical properties of miPEP165a. Figure S3. Quantification of miPEP165a-FAM uptake in Arabidopsis roots. Figure S4. MβCD impairs the miPEP165a-FAM entry in the Arabidopsis root cap/meristematic zone.

## Abbreviations

AGO1	Argonaute 1
AP2	adaptor protein 2
CAN/AtHB15	CORONA
CHC	clathrin heavy chain
CLC	clathrin light chain
CME	clathrin-mediated endocytosis
DCL1	dicer-like1
FAM	5-carboxyfluorescein
HB8	homeobox gene 8
HD-ZIP III	class III homeodomain-leucine zipper
MβCD	methyl-β-cyclodextrin
miPEP	miRNA-encoded peptide
miRNA	micro-RNA
MS	Murashige and Skoog medium
PHB	PHABULOSA
PHV	PHAVOLUTA
PIP2;1	plasma membrane intrinsic protein 2
pri-miRNA	primary-microRNA
pre-miRNA	precursor-microRNA
RbohD	respiratory burst oxidase protein D
REM	remorin
REV	REVOLUTA
TyrA23	Tyrphostin A23.
