# Supplementary material for: Internalization of miPEP165a into Arabidopsis Roots Depends on both Passive Diffusion and Endocytosis-Associated Processes
Source: Int J Mol Sci. 2020 Mar 25;21(7):2266. doi: 10.3390/ijms21072266 (PMC7178249; doi:10.3390/ijms21072266)
Supplement: Supplementary file 1 [file ijms-21-02266-s001.pdf]

Figure S1

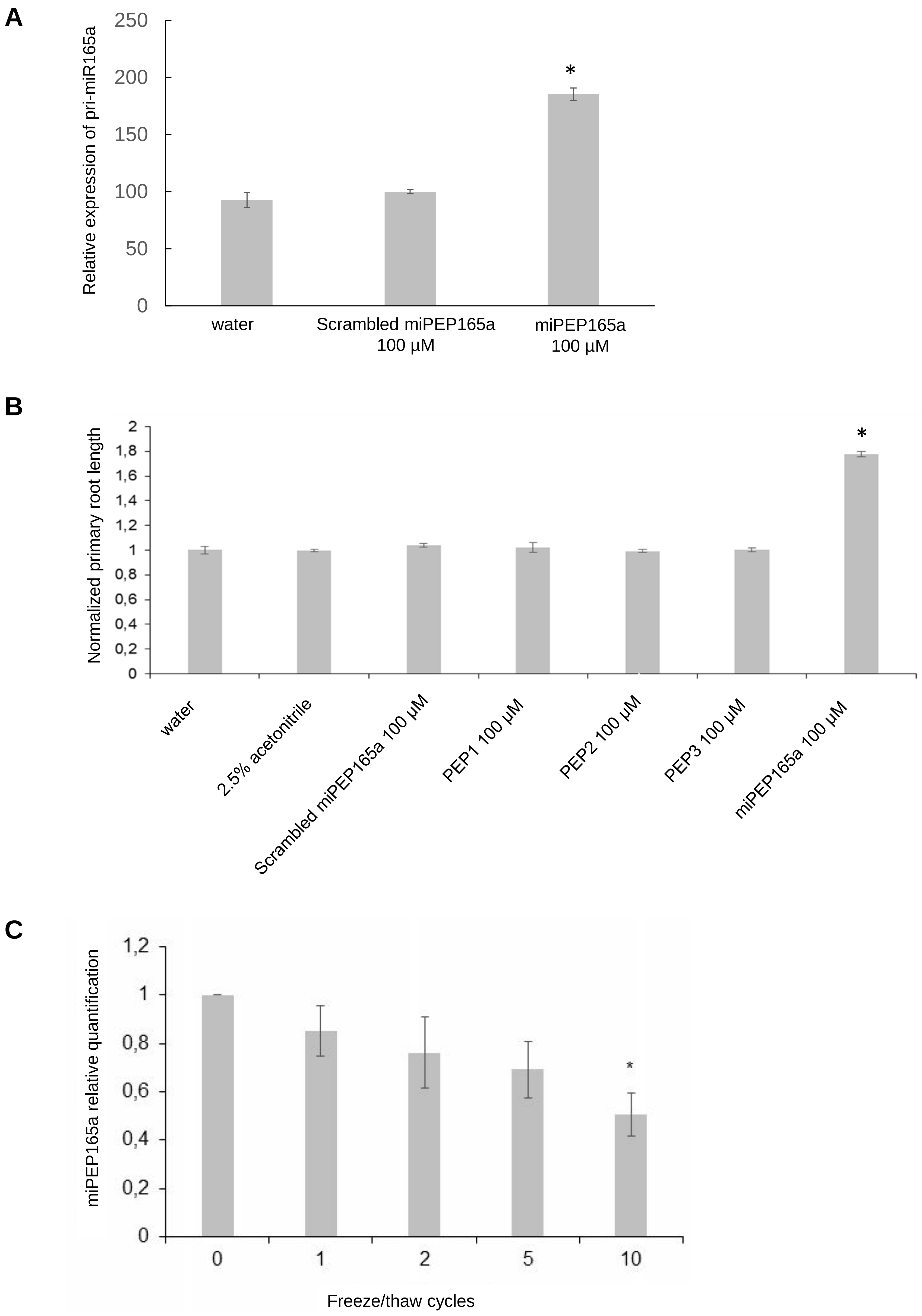

Figure S1

Effect of miPEP165a and importance of its stability. (**A**) Expression by RT-qPCR of pri-miR165a in *Arabidopsis* seedlings treated for 24h either with water or synthetic scrambled miPEP165a or miPEP165a at 100 µM. The error bars represent SEM of three biological experiments (n ≈ 10 seedlings). Statistical analysis was performed using a Kruskal–Wallis test (*, $P < 0.05$). (**B**) Effects of the different controls on primary root length compared to the miPEP165a. *Arabidopsis* seedlings were treated daily for 4 days with water, 2.5% acetonitrile, scrambled miPEP165a, irrelevant peptide (PEP1, PEP2, PEP3) and miPEP165a at 100 µM. Root lengths were normalized compared to water condition. Three biological experiments have been performed. Error bars indicate SEM and statistical analyses were performed using a t-test (n ≈ 80; *, $P < 0.05$). (**C**) Effect of freeze/thaw cycles on degradation of miPEP165a. Five nanomoles of peptides were frozen/thawed several times and blotted with an antibody recognizing miPEP165a. Histograms show the mean of the quantification of 6 independent western blots. Quantification was performed using ImageJ. Error bars represent SEM and asterisk indicates a significant difference between the treatment condition and the control according to the Kruskal-Wallis test ($P < 0.05$).

Figure S2

A

B

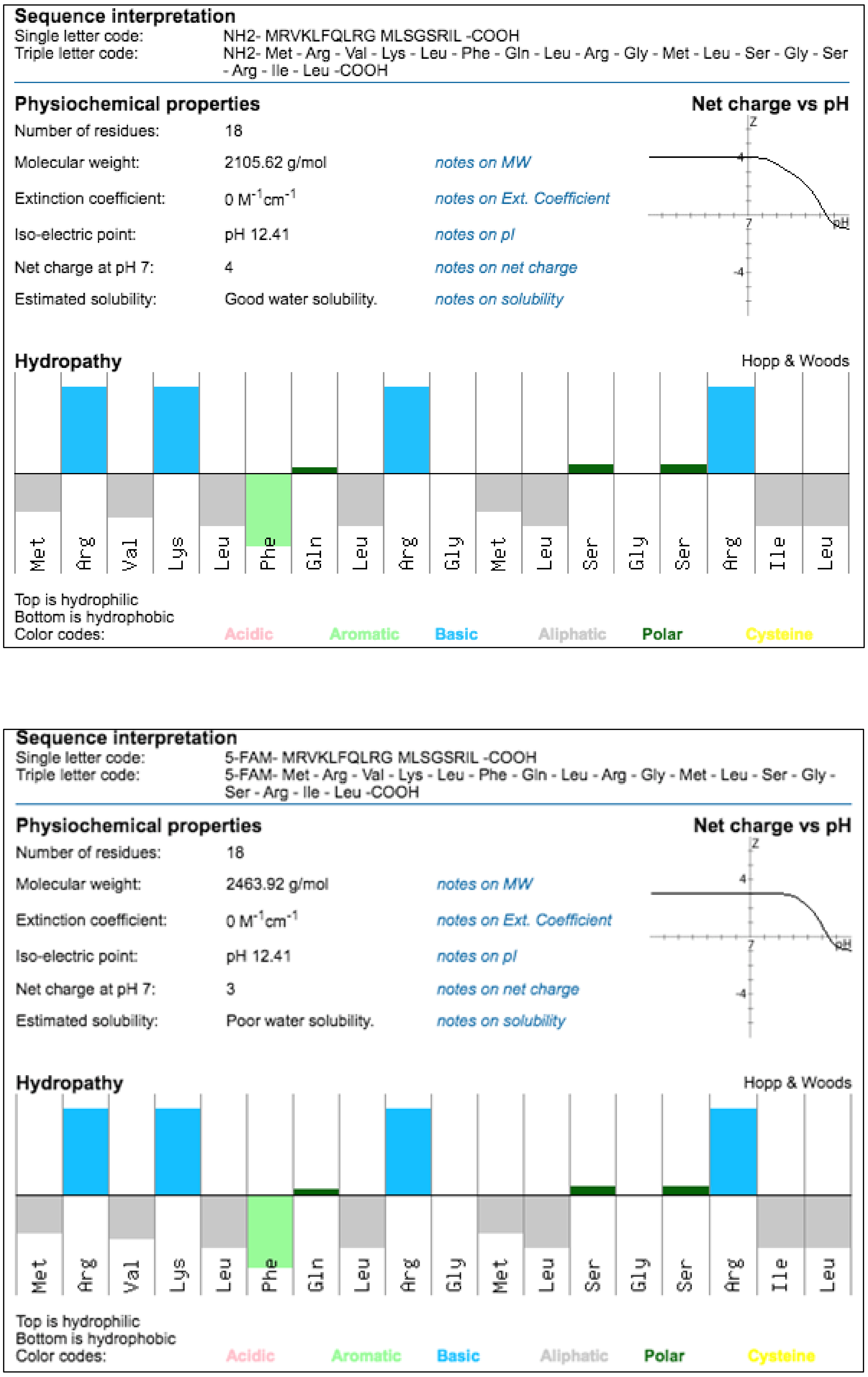

Figure S2
Physiochemical properties of miPEP165a (A) and miPEP165a-FAM (B).
Physiochemical properties were calculated using the software peptide calculator (PepCal, https://pepcalc.com/).

Figure S3

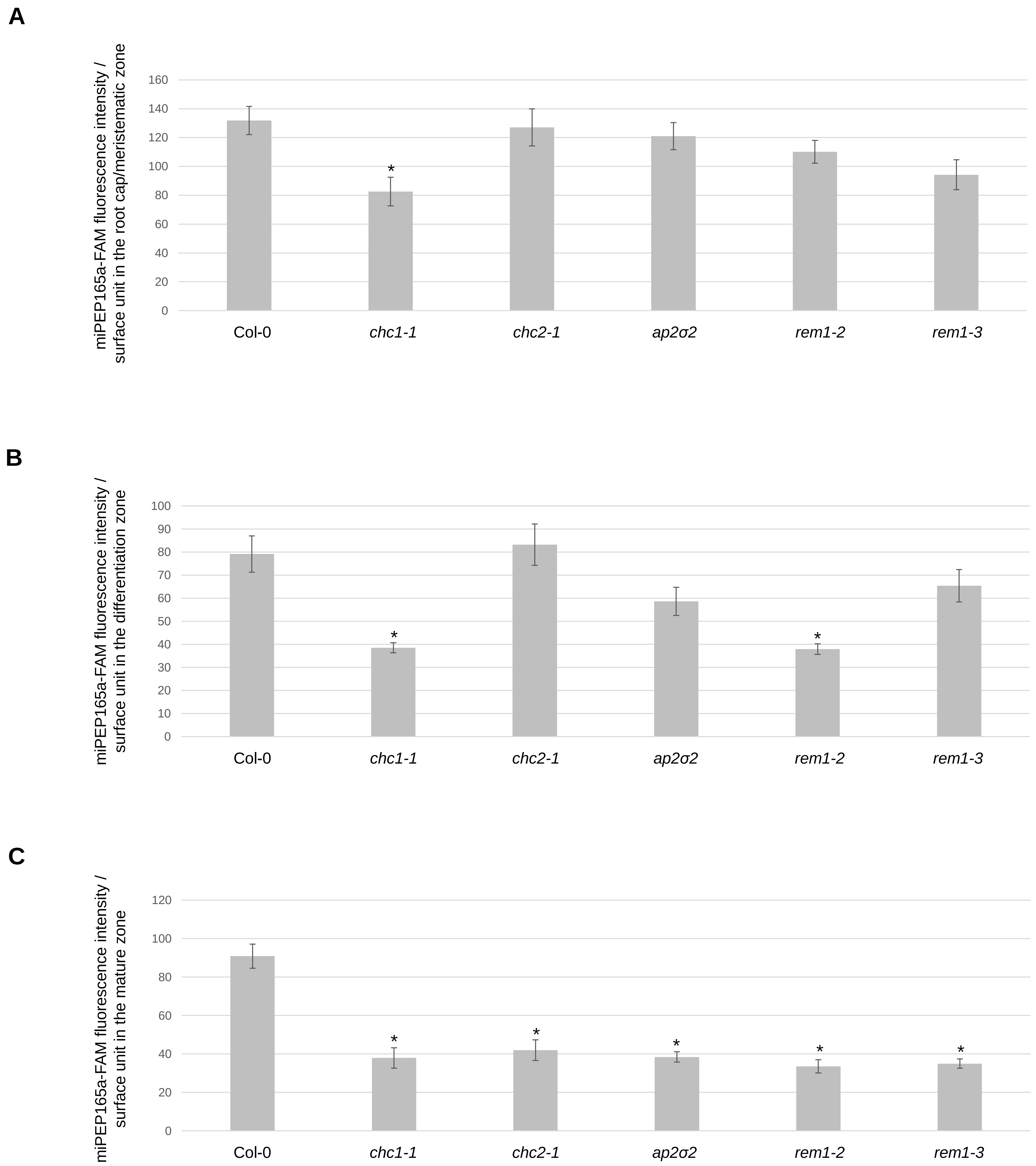

Figure S3

Quantification of miPEP165a-FAM uptake in *Arabidopsis* roots. Fluorescence intensity in Figure 4 was quantified per surface unit for wild-type and mutant plants in the root cap/meristematic zone (**A**), differentiation zone (**B**) and mature zone (**C**) using ImageJ software. Experiments were performed at least twice with similar results (n > 15 seedlings). Error bars represent SEM. Significant differences between wild-type and mutant plants were indicated by *, $P < 0.01$ (t-test).

Figure S4

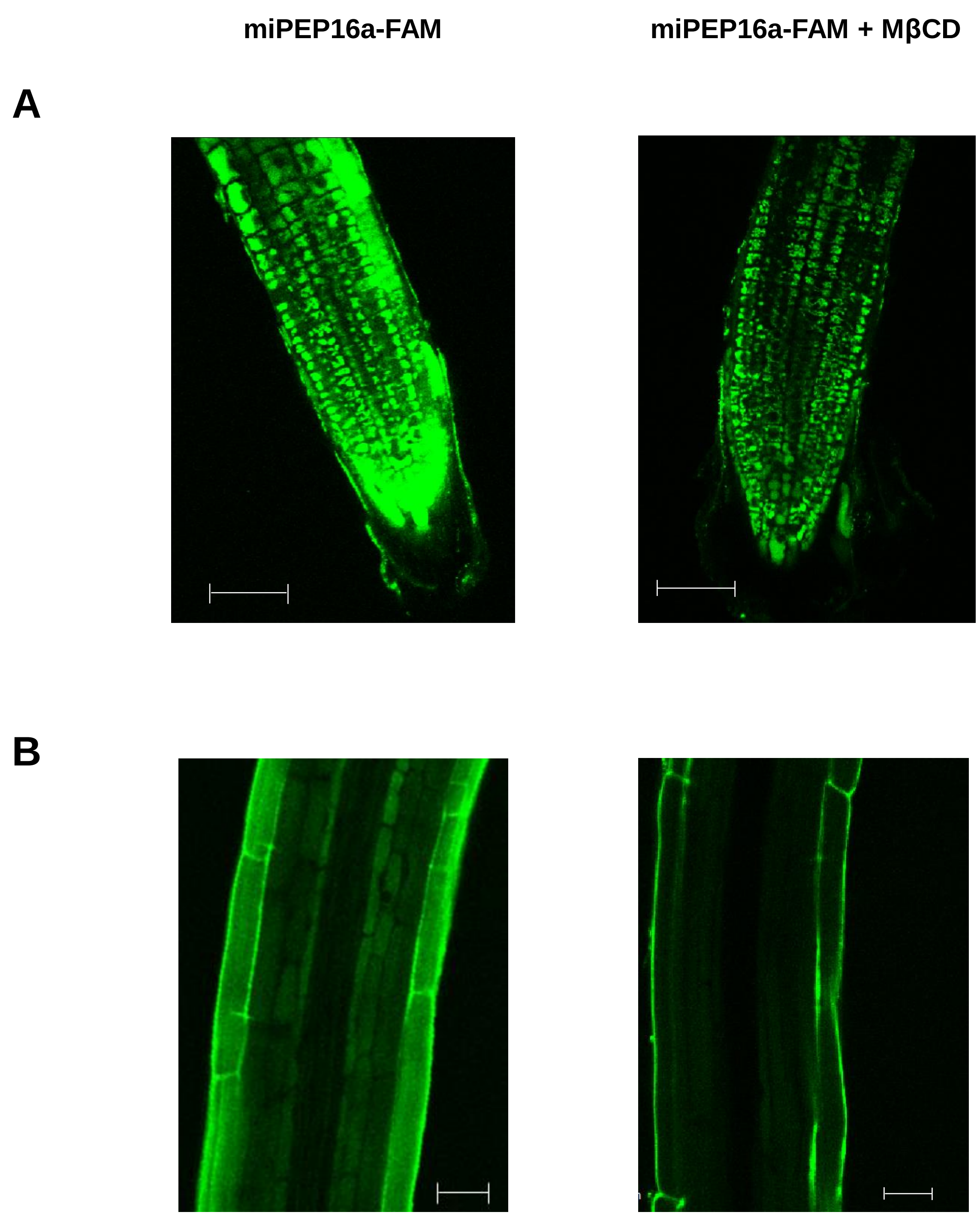

Figure S4

MβCD impairs the miPEP165a-FAM entry in the *Arabidopsis* root cap/meristematic zone (A) and in the mature zone (B). Confocal images are representative of three independent experiments. Scale bar = 50 µm (root cap/meristematic zone) or 25 µm (mature zone).
